# Deactivation of excitatory neurons in the prelimbic cortex via Cdk5 promotes pain sensation and anxiety

**DOI:** 10.1038/ncomms8660

**Published:** 2015-07-16

**Authors:** Guo-Qiang Wang, Cheng Cen, Chong Li, Shuai Cao, Ning Wang, Zheng Zhou, Xue-Mei Liu, Yu Xu, Na-Xi Tian, Ying Zhang, Jun Wang, Li-Ping Wang, Yun Wang

**Affiliations:** 1 Neuroscience Research Institute and Department of Neurobiology, Key Laboratory for Neuroscience of Ministry of Education and Neuroscience, National Health and Family Planning Commission, Peking University, Beijing 100191, China.; 2 Shenzhen Key Lab of Neuropsychiatric Modulation, CAS Center for Excellence in Brain Science, Shenzhen Institutes of Advanced Technology, Chinese Academy of Sciences, Shenzhen 518055, China.; 3 Department of Anatomy and Histology, School of Basic Medical Sciences, Peking University, Beijing 100191, China.; 4 PKU-IDG/McGovern Institute for Brain Research, Peking University, Beijing 100871, China.

## Abstract

The medial prefrontal cortex (mPFC) is implicated in processing sensory-discriminative and affective pain. Nonetheless, the underlying mechanisms are poorly understood. Here we demonstrate a role for excitatory neurons in the prelimbic cortex (PL), a sub-region of mPFC, in the regulation of pain sensation and anxiety-like behaviours. Using a chronic inflammatory pain model, we show that lesion of the PL contralateral but not ipsilateral to the inflamed paw attenuates hyperalgesia and anxiety-like behaviours in rats. Optogenetic activation of contralateral PL excitatory neurons exerts analgesic and anxiolytic effects in mice subjected to chronic pain, whereas inhibition is anxiogenic in naive mice. The intrinsic excitability of contralateral PL excitatory neurons is decreased in chronic pain rats; knocking down cyclin-dependent kinase 5 reverses this deactivation and alleviates behavioural impairments. Together, our findings provide novel insights into the role of PL excitatory neurons in the regulation of sensory and affective pain.

## Abstract (web summary)

The medial prefrontal cortex (mPFC) is implicated in pain regulation, yet the underlying mechanisms are poorly understood. Here the authors establish a critical role for mPFC in regulating pain sensation and pain-related anxiety, mediated by activation of the cyclin-dependent kinase 5 signalling pathway.

## Graphical abstract

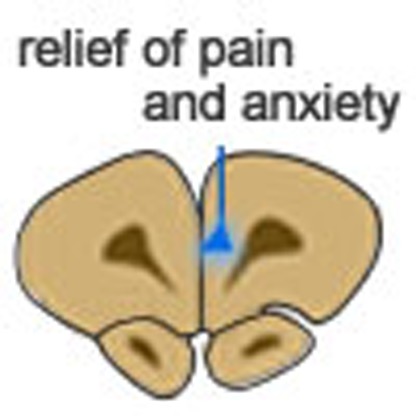

According to epidemiological data of Europe, one-fifth of the world population suffers from chronic pain^1^. During acute pain, physiological and psychological processes, such as perception, cognition, emotion, motivation and memory, adapt to prevent further damage. However, for chronic pain, such changes are maladaptive; thus, chronic pain is typically accompanied by comorbidities, such as hemi-inattention, deficits in cognition and memory, mood disorders and decreased motivation^2^. Clinically, patients with chronic pain are at a heightened risk for developing anxiety^3^,^4^. Likewise, patients with anxiety express more pain complaints and typically exhibit greater perceptual and cognitive impairments^5^. The interactions between pain and anxiety have made the evaluation and treatment of chronic pain clinically challenging. Moreover, the specific neural circuits involved in chronic pain-induced anxiety are not yet fully understood.

In chronic pain, nociceptors trigger an increased rate of action potentials (APs) and subsequently convey pain signals to several brain areas that are thought to be involved in the initiation of pain perception and anxiety^6^. Functional imaging studies suggest the involvement of primary somatosensory cortex (S1) and secondary somatosensory cortex (S2) in processing the sensory-discriminative component of pain and the anterior cingulate cortex (ACC) and prefrontal cortex (PFC) in processing the affective-motivational component of pain^7^. Previous studies largely focus on the role of the ACC in affective responses to pain. Long-term potentiation (LTP) in the ACC is thought to maintain chronic pain, and the disruption of LTP in the ACC alleviates chronic pain^8^. Chronic pain and anxiety are both associated with the occlusion of a presynaptic form of LTP in the ACC, suggesting that the two forms of LTP in the ACC may mediate the interaction between anxiety and chronic pain^9^.

In addition to the ACC, recent studies address the importance of the medial PFC (mPFC) in various cognitive and emotional processes, and pain-related perceptions^10^. Functional and morphological abnormalities in the mPFC are present in a chronic pain animal model^11^. Moreover, the mPFC exhibits increased blood flow following an acute nociceptive stimulation^12^. Both background and evoked activity of mPFC neurons is decreased in an arthritis chronic pain model^13^,^14^ and it has been speculated that the progression of pain from acute to chronic is accompanied by a decrease in mPFC activity^15^. In addition to its role in pain sensation, the mPFC has also been implicated in anxiety. Functional imaging studies indicate that the mPFC is hypoactive in anxiety patients^16^. The mPFC is also involved in anxiety-like behaviours in animal models of stress^17^,^18^. Based on these evidence, we hypothesized that the mPFC is critical in the initiation of pain-related anxiety and the modulation of pain sensation.

In this study, we use a chronic inflammatory rodent pain model to examine the role of mPFC in chronic pain and anxiety. We show that chemically lesioning the PL, a sub-region of the mPFC, contralateral but not ipsilateral to the inflamed hind paw in rats attenuates anxiety-like behaviours and heat hyperalgesia, and that the intrinsic excitability of layer 2/3 excitatory neurons in the contralateral PL is decreased. When optogenetically activated, contralateral PL neurons exert analgesic and anxiolytic effects in chronic inflammatory pain mice; on the other hand, optogentically inhibiting them has an anxiogenic effect in naive mice. We subsequently show that the decreased activation of excitatory neurons in the contralateral PL of chronic pain rats is due to activation of cyclin-dependent kinase 5 (Cdk5), and that knockdown of Cdk5 reverses the deactivation of these excitatory neurons and attenuates anxiety-like behaviours and heat hyperalgesia. Together, these findings provide new insights into emotional and pain treatment in clinical chronic pain management.

## Results

### Lesion of contralateral PL attenuates pain and anxiety

**Figure 1:**
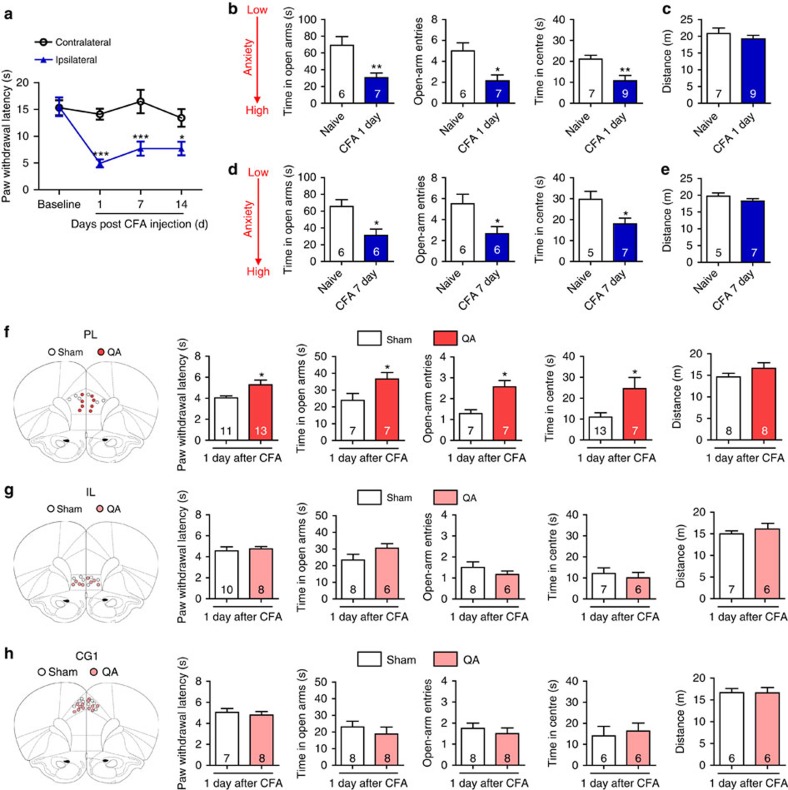
Bilateral lesions of the PL attenuate CFA-induced heat hyperalgesia and anxiety-like behaviours. (**a**) Paw withdrawal latency of the contralateral and ipsilateral hind paws after CFA injection (*n*=5 animals, *P<0.05, ***P<0.001, two-way ANOVA with Bonferroni post tests). (**b**) Elevated plus maze (left and middle, *n*=6, 7 animals, *P<0.05, **P<0.01, two-tailed *t*-test) and open field test (right, *n*=7, 9 animals, **P<0.01, two-tailed *t*-test) 1 day after CFA injection. (**c**) Locomotor activity 1 day after CFA injection (*n*=7, 9 animals, two-tailed *t*-test). (**d**) Elevated plus maze (left and middle, *n*=6, 6 animals, *P<0.05, two-tailed *t*-test) and open field test (right, *n*=5, 7 animals, *P<0.05, two-tailed *t*-test) 7 days after CFA injection. (**e**) Locomotor activity 7 days after CFA injection (*n*=5, 7 animals, two-tailed *t*-test). (**f**–**h**) Paw withdrawal latency, elevated plus maze, open field test and locomotor activity of the CFA rats after bilateral lesion of the PL, IL or CG1 by quinolinic acid, respectively (*P<0.05, two-tailed *t*-test). The data are presented as mean±s.e.m.

To address the role of the mPFC in the modulation of pain sensation and pain-induced anxiety, we used a complete Freund’s adjuvant (CFA)-induced chronic inflammatory pain model. Rats exhibited persistent heat hyperalgesia in the ipsilateral hind paw compared with the contralateral hind paw at 1, 7 and 14 days after CFA was injected in the left hind paw (Fig. 1a). Chronic pain rats exhibited both decreased time in the open arms and decreased open-arm entries of the elevated plus maze (EPM) as well as decreased time in the centre of the open field test (OFT) both 1 and 7 days after CFA injection (Fig. 1b,d); in contrast, general locomotor ability was unaffected (Fig. 1c,e). These results suggest that CFA-induced pain symptoms and anxiety-like behaviours develop and rapidly reach a plateau, providing a well-defined model to study pain sensation and pain-related anxiety-like behaviours.

**Figure 2:**
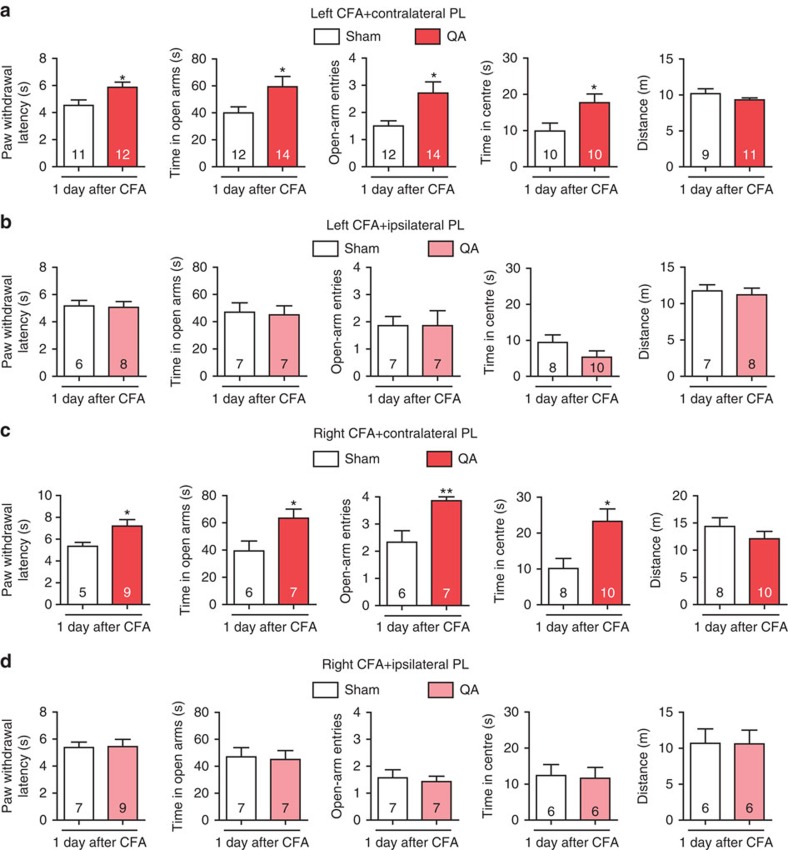
Contralateral lesion of the PL exerts analgesic and anxiolytic effects. (**a**, **b**) Paw withdrawal latency, elevated plus maze, open field test and locomotor activity of the rats with CFA injected in the left hind paw after contralateral or ipsilateral lesion of the PL by QA, respectively (*P<0.05, two-tailed *t*-test). (**c**, **d**) Paw withdrawal latency, elevated plus maze, open field test and locomotor activity of the rats with CFA injected in the right hind paw after contralateral or ipsilateral lesion of the PL by QA, respectively (*P<0.05, **P<0.01, two-tailed *t*-test). The data are presented as mean±s.e.m.

To pinpoint the specific sub-region(s) in the mPFC that are responsible for pain sensation and pain-induced anxiety, we bilaterally damaged the three sub-regions, the cingulate cortex area 1 (CG1), PL and infralimbic cortex (IL), respectively, by micro-infusion of quinolinic acid (QA), a potent neurotoxic compound. The injection of QA induced the neuronal loss suggested by the NeuN immunostaining (Supplementary Fig. 1). Following bilateral lesion of the PL, rats exhibited increased paw withdrawal latencies (PWL), increased time in the open arms and centre and increased open-arm entries (Fig. 1f), whereas bilateral lesions of the CG1 or IL had no effect on the PWL, EPM or OFT performances (Fig. 1g,h). Bilateral lesion of the PL in naive rats attenuated anxiety-like behaviours in the EPM and OFT without affecting the PWL (Supplementary Fig. 2). These findings suggest that the PL rather than the CG1 or IL may be responsible for the modulation of pain sensation and the initiation of anxiety-like behaviours induced by CFA. Considering pain signalling is conveyed to the contralateral cortex from the level of spinal cord, we next examined the consequences of individually damaging the contralateral and ipsilateral PL on the modulation of pain sensation and pain-related anxiety-like behaviours. In rats with contralateral PL damage, the PWL was augmented and the time in the open arms and open-arm entries in the EPM as well as the time in centre of the OFT increased (Fig. 2a). In contrast, ipsilateral lesion of the PL did not affect heat hyperalgesia or anxiety-like behaviours (Fig. 2b). It has been reported that pain-associated affect is more pronounced when pain is localized in the left side of the body^19^. To exclude the possibility of cortical function lateralization, we damaged the right hind paw with a CFA injection and lesioned the left (contralateral) PL and obtained similar results as those observed following CFA injection in the left hind paw and right PL lesion (Fig. 2c,d). These results suggest that the contralateral but not ipsilateral PL has a vital role in the modulation of pain sensation and pain-related anxiety-like behaviours.

### Contralateral PL excitatory neurons are deactivated post CFA

**Figure 3:**
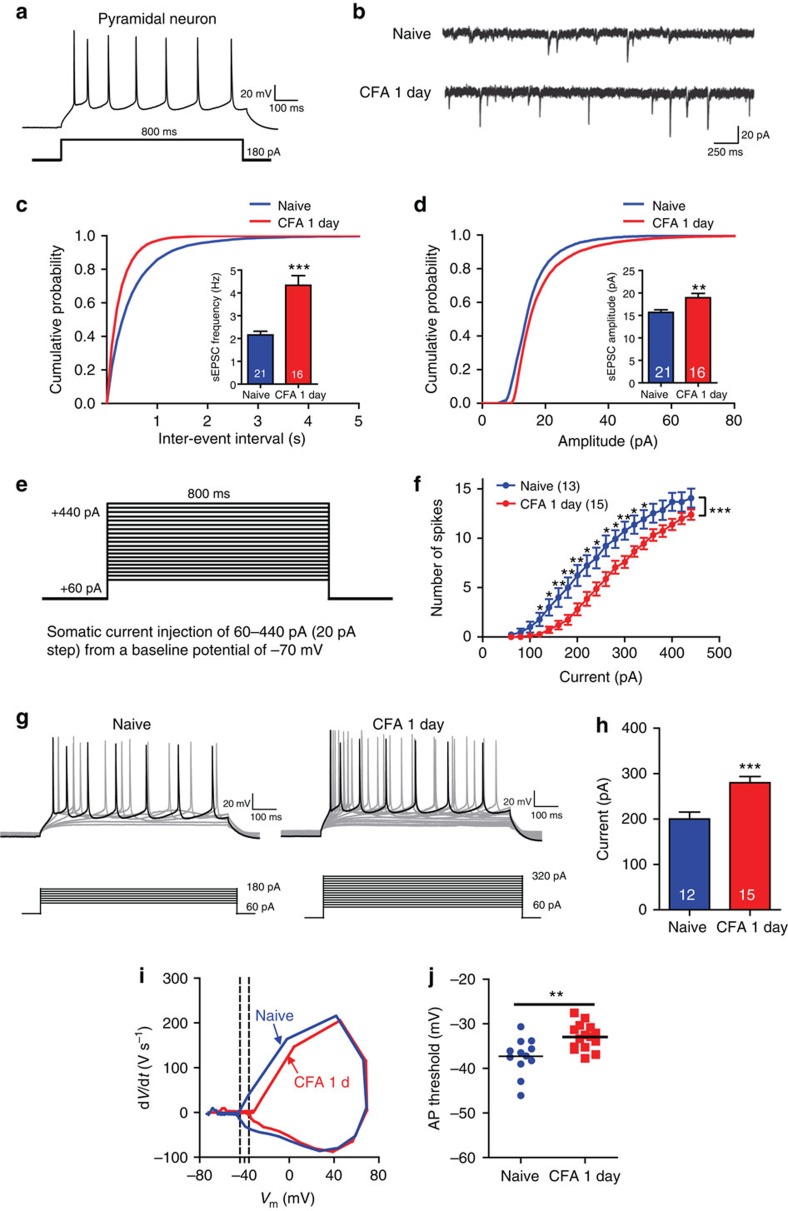
The intrinsic excitability of layer 2/3 neurons is decreased in the contralateral PL 1 day after CFA injection. (**a**) Current-clamp recordings to identify excitatory neurons in the PL. (**b**) Representative sEPSC recording traces from the naive rats and the rats 1 day after CFA. Scale bars, 20 pA and 250 ms. (**c**, **d**) Cumulative probabilities (***P<0.001, Kolmogorov–Smirnov two sample test) and average sEPSC frequencies and amplitudes from the naive rats (frequency: 2.154±0.1671 Hz, amplitude: 15.67±0.5745, pA, *n*=21 neurons) and the rats 1 day after CFA (frequency: 4.3324±0.4222 Hz, amplitude: 18.93±0.9648, pA, *n*=16 neurons, **P<0.01, ***P<0.001, two-tailed *t*-test). (**e**) Recording paradigm of the excitatory neurons in the PL. (**f**) Number of spikes induced by injected currents in the contralateral PL excitatory neurons from the naive rats and the rats 1 day after CFA (*n*=13, 15 neurons, *P<0.05, **P<0.01, ***P<0.001, two-way ANOVA with Bonferroni post tests). (**g**) Examples of the AP responses to positive current steps recorded from pyramidal neurons from the naive rats and the rats 1 day after CFA. (**h**) The current required to drive APs at a frequency of 8.75 Hz from naive rats (200±15.37 pA, *n*=12 neurons) and the rats 1 day after CFA (280±13.66 pA, *n*=15 neurons; ***P<0.001, two-tailed *t*-test). (**i**) Phase plots of the APs from the naive rats and the rats 1 day after CFA evoked by brief current injections. (**j**) Plot diagram summary of the AP thresholds from the naive rats (−37.29±1.185 mV, *n*=12 neurons) and the rats 1 day after CFA (−32.95±0.7555, mV, *n*=15 neurons; **P<0.01, two-tailed *t*-test). The data are presented as mean±s.e.m.

To address how the contralateral PL is involved in CFA-induced heat hyperalgesia and pain-related anxiety, we first tested whether chronic pain alters the physiology of PL neurons by recording spontaneous excitatory post-synaptic currents (sEPSCs) in layer 2/3 neurons in PL using whole-cell patch clamp recordings. Excitatory pyramidal neurons in naive rats exhibited spike frequency adaption in response to a depolarizing current pulse (Fig. 3a). At 1 day post CFA injection, excitatory layer 2/3 neurons in the contralateral PL exhibited increased sEPSC frequencies and amplitudes compared with those of naive rats (Fig. 3b–d). These results, which were also confirmed by differences in the cumulative probabilities of sEPSC frequency and amplitude (Fig. 3c,d), are consistent with previous work using a spared nerve injury chronic pain model^11^.

We subsequently determined whether CFA injections altered the intrinsic excitability of contralateral PL neurons by examining biophysical characteristics of APs. APs were evoked by superimposed positive current steps (Fig. 3e) from a baseline potential of −65 mV. A rightward shift in the input–output curve appeared 1 day after CFA injection, and significant differences were observed from current injections ranging from 180 to 300 pA (Fig. 3f). Examination of input–output curves indicated that fewer APs were elicited by depolarizing current steps at 1 day following CFA, compared with recordings obtained from naive rats. These results are consistent with previous results obtained in an arthritis pain model^14^. The current required to drive spike firing at 8.75 Hz was substantially greater at 1 day following CFA compared with that required to drive spike firing in naive rats (Fig. 3g,h). To analyse this further, the firing threshold of the first AP at 8.75 Hz was determined by a phase plot (Fig. 3i). The neurons taken from rats 1 day after CFA injection have a more depolarized firing threshold compared with the neurons in naive rats (Fig. 3j). Additionally, the resting membrane potential was slightly more positive in neurons taken from rats 1 day following CFA injection as compared with that observed in naive controls, whereas no significant difference in input resistance was identified between groups (Supplementary Figs 3a,b). These results suggest that although glutamatergic synaptic transmission in the contralateral PL is enhanced, the intrinsic excitability of excitatory neurons is decreased at the single-neuron level 1 day following CFA injection.

### Activation of PL excitatory neurons reduces pain and anxiety

**Figure 4:**
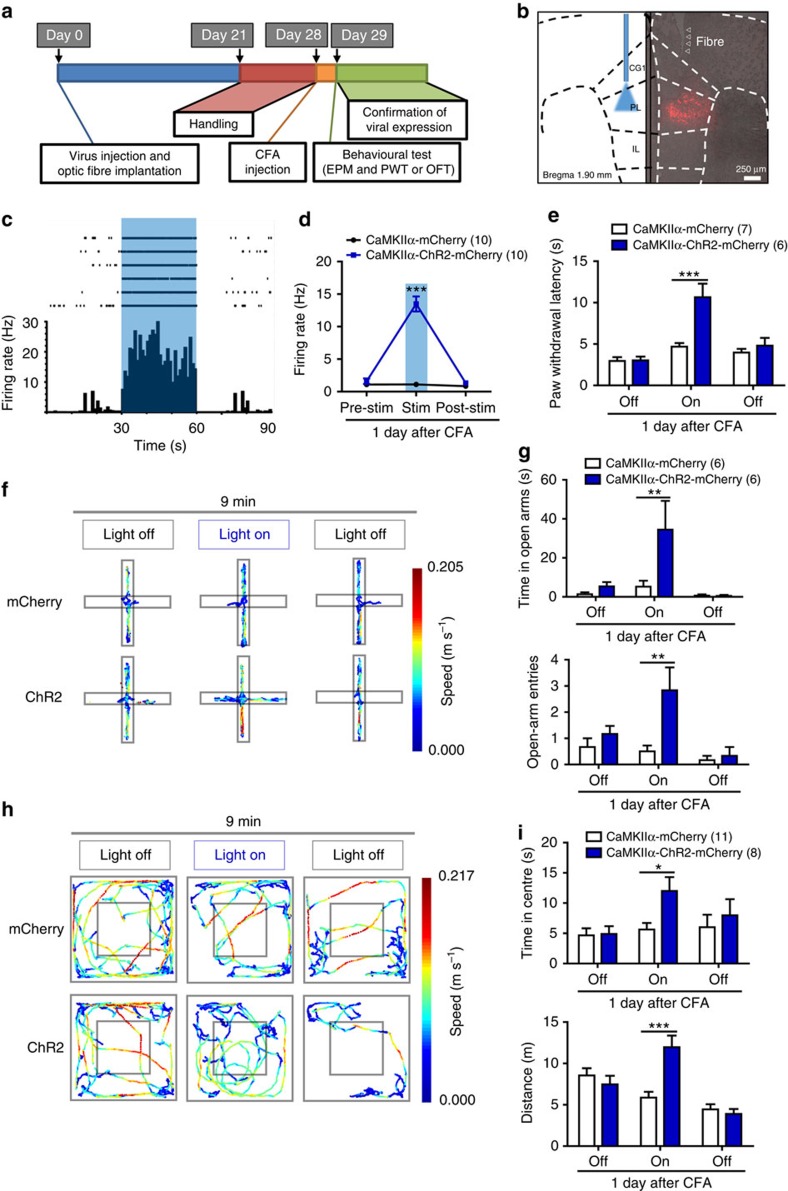
Optogenetic activation of contralateral PL excitatory neurons attenuates CFA-induced heat hyperalgesia and anxiety-like behaviours. (**a**) Schematic diagram of the experiment. One group animals were tested for EPM followed by paw withdrawal latency test. Another group animals were tested for OFT. (**b**) ChR2 expression in the PL excitatory neurons after viral injection. Strong staining is shown in the PL (Bregma 1.90 mm). (**c**, **d**) Multiple channel recordings of the firing rate of the contralateral PL excitatory neurons in the CFA mice infected with AAV-CaMKIIα-mCherry and AAV-CaMKIIα-ChR2-mCherry virus before, with and after 20 Hz, 6–9 mW 473-nm light photostimulation (*n*=10, 10 neurons, ***P<0.001, two-way ANOVA with Bonferroni post tests). (**e**) Paw withdrawal latency in the CFA mice of AAV-CaMKIIα-mCherry or AAV-CaMKIIα-ChR2-mCherry virus injection with 473 nm blue light off-on-off stimulation (*n*=7, 6 animals, ***P<0.001, two-way ANOVA with Bonferroni post tests). (**f**, **g**) Schematic and animal heat traces of the elevated plus maze with 3 min light-off, 3 min light-on and 3 min light-off (*n*=6, 6 animals, **P<0.01, two-way ANOVA with Bonferroni post tests). (**h**, **i**) Schematic and animal heat traces of the open field test with 3 min light-off, 3 min light-on and 3 min light-off (*n*=11, 8 animals, *P<0.05, ***P<0.001, two-way ANOVA with Bonferroni post tests). The data are presented as mean±s.e.m.

To determine whether the deactivation of excitatory neurons in the contralateral PL is required for pain-induced behavioural impairments, we used optogenetics techniques that enable activation or inhibition of cell-type-specific neurons. Since the optogenetic experiments were performed in C57BL/6 mice, we first confirmed that CFA also induced anxiety-like behaviours in C57BL/6 mice in EPM and OFT (Supplementary Fig. 4). To enable optical excitation of PL excitatory neurons, we infected PL excitatory neurons using an adeno-associated viral vector serotype 5 (AAV5) carrying channelrhodopsin-2 (ChR2) under control of the CaMKIIα promoter (Fig. 4b). We used multi-channel recordings to determine effective illumination parameters. We found that 25-ms pulses of 473 nm blue light presented at 20 Hz with an intensity of 6–9 mW increased the firing rate of excitatory neurons from 1.59±0.42 to 13.49±1.16 Hz in AAV5-CaMKIIα-ChR2-mCherry-infected CFA mice (Fig. 4c,d). Four weeks after viral infection and optic fibre implantation, the mice were tested in the EPM and OFT, using a 3-min light off-on-off paradigm (Fig. 4a). To test whether anxiety-like behaviours were related to the deactivation of contralateral PL excitatory neurons, we compared behaviour of AAV5-CaMKIIα-ChR2-mCherry-infected mice with that of a control group of mice infected with a control virus (AAV5-CaMKIIα-mCherry) but receiving identical optical stimulation. Photostimulation of contralateral PL excitatory neurons increased the time spent in the open arms and open-arm entries of the EPM (Fig. 4f,g) and the time in the centre of the OFT (Fig. 4h,i). However, activation of the contralateral PL excitatory neurons also increased the travel distance in the OFT (Fig. 4i). We further tested whether the contralateral PL was involved in the modulation of pain sensation. The PWL was measured every 3 min (for a total of four repeats) under 20 Hz 473-nm blue light photostimulation, and the mean PWL was calculated. The activation of contralateral PL excitatory neurons significantly increased the PWL, which suggests an alleviation of heat hyperalgesia (Fig. 4e). As 1 day post CFA injection may not be considered chronic, we further tested a later time-point post CFA injection. Activation of contralateral PL excitatory neurons also exerted analgesic and anxiolytic effects 7 days after CFA injection (Supplementary Fig. 5). However, the activation of the excitatory neurons in the ipsilateral PL, contralateral CG1 or IL did not affect anxiety-like behaviours or heat hyperalgesia (Supplementary Figs 6–8), confirming the specific role of the contralateral PL. Furthermore, activation of excitatory neurons in the ipsilateral PL also increased the distance travelled in the OFT (Supplementary Fig. 5d), suggesting that the relief of anxiety-like behaviours via the activation of contralateral PL excitatory neurons is not a result of the increase in travel distance.

Previous studies indicate that the mPFC is involved in anxiety-like behaviours^17^,^18^. To exclude the possibility that the mPFC regulates anxiety relief independent of pain state, we optogenetically activated PL excitatory neurons unilaterally in naive mice, and no obvious changes in anxiety-like behaviours were observed (Supplementary Figs 9a–d), indicating that the relief of anxiety-like behaviours in the CFA mice was pain-related. However, the PWL was increased in naive mice after PL excitatory neuron activation (Supplementary Fig. 9e). These results suggest that the activation of contralateral PL excitatory neurons attenuates anxiety-like behaviours and heat hyperalgesia induced by CFA.

### Inhibition of PL excitatory neurons initiates anxiety

**Figure 5:**
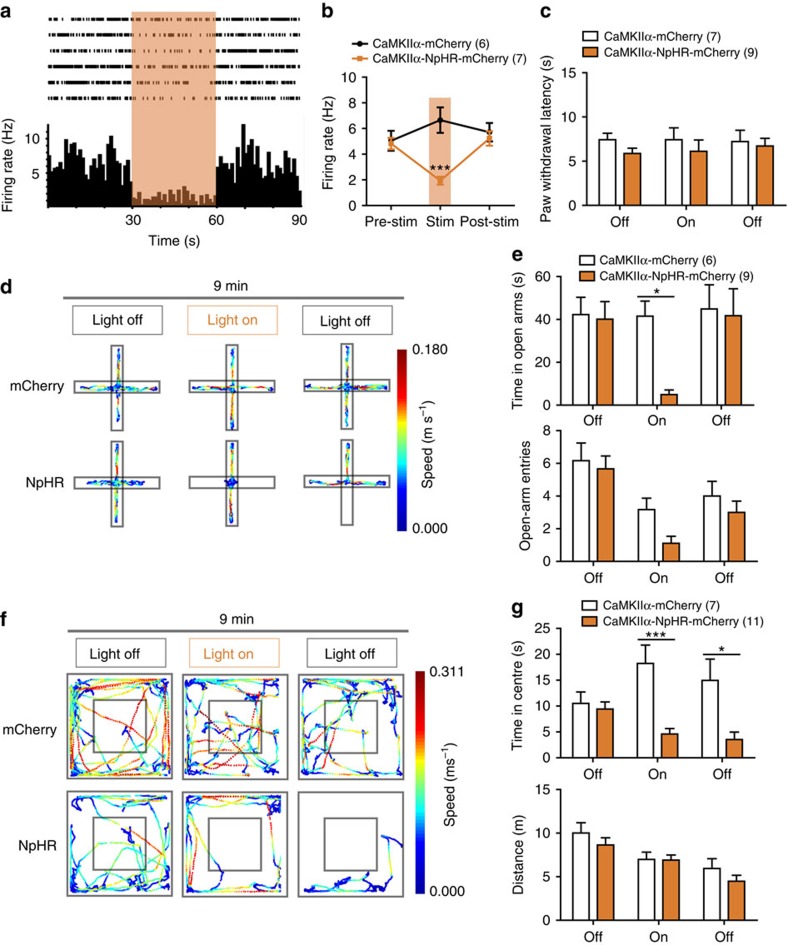
Optogenetic inhibition of PL excitatory neurons elicits anxiety-like behaviours. (**a**, **b**) Multiple channel recordings of the firing rate of the PL excitatory neurons in the mice infected with AAV-CaMKIIα-mCherry and AAV-CaMKIIα-NpHR-mCherry virus before, with and after continuous 6–9 mW 593 nm light photostimulation (*n*=6, 7 neurons, ***P<0.001, two-way ANOVA with Bonferroni post tests). (**c**) Paw withdrawal latency in the naive mice of the AAV-CaMKIIα-mCherry or AAV-CaMKIIα-NpHR-mCherry virus injection with 593 nm yellow light off-on-off stimulation (*n*=7, 9 animals, two-way ANOVA with Bonferroni post tests). (**d**, **e**) Schematic and animal heat traces of the elevated plus maze with 3 min light-off, 3 min light-on and 3 min light-off (*n*=6, 9 animals, *P<0.05, two-way ANOVA with Bonferroni post tests). (**f**, **g**) Schematic and animal heat traces of the open field test with 3 min light-off, 3 min light-on and 3 min light-off (*n*=7, 11 animals, *P<0.05, **P<0.01, two-way ANOVA with Bonferroni post tests). The data are presented as mean±s.e.m.

To ascertain the role of contralateral PL excitatory neurons in the initiation of anxiety-like behaviours, we unilaterally transduced PL excitatory neurons with AAV5 that carried the third generation of halorhodopsin (NpHR3.0) under the control of the CaMKIIα promoter. NpHR3.0 is a light-driven chloride pump that hyperpolarizes neurons in response to 593 nm of yellow light. Continuous 593 nm yellow light illumination with an intensity of 6–9 mW decreased the firing rate of the excitatory neurons from 4.82±0.44 to 1.94±0.31 Hz (Fig. 5a,b). The unilateral inhibition of PL excitatory neurons significantly decreased the time spent in the open arms of the EPM (Fig. 5d,e) and the time in the centre of the OFT (Fig. 5f,g). Open-arm entries in the EPM tended to be fewer in number when PL excitatory neurons were unilaterally inhibited (Fig. 5e). However, the unilateral inhibition of PL excitatory neurons did not affect the PWL (Fig. 5c), suggesting that PL excitatory neurons exert different roles in physiological and pathophysiological states.

### Cdk5 is activated in the contralateral PL post CFA

**Figure 6:**
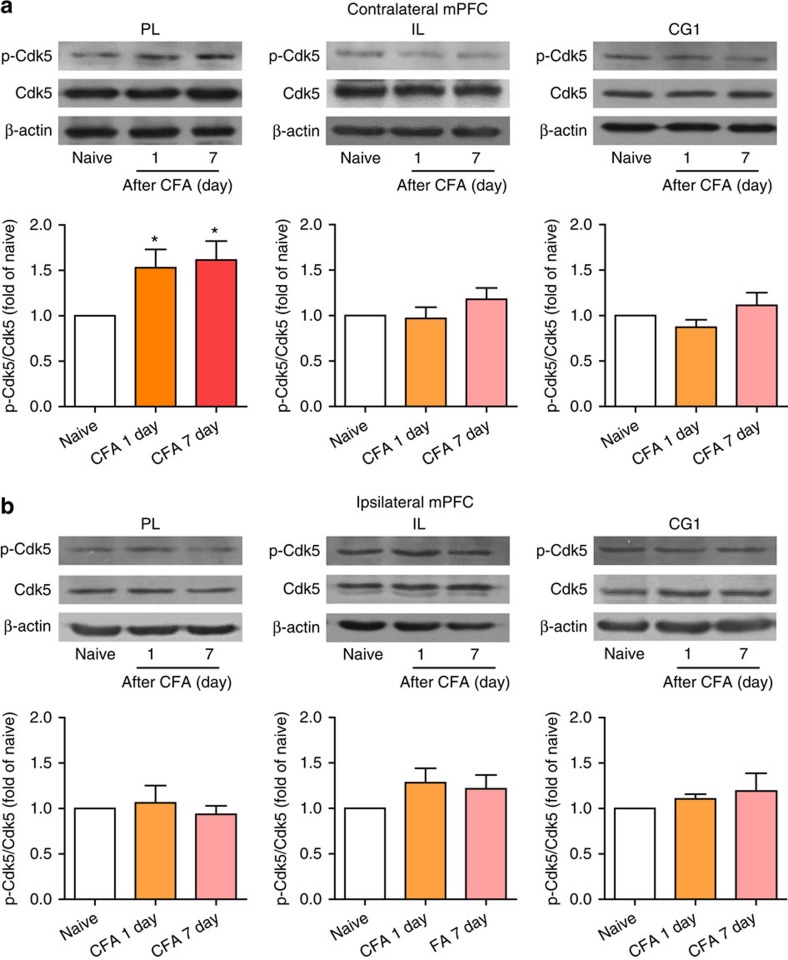
Cdk5 is activated in the contralateral PL after CFA injection. (**a**, **b**) Time course of the p-Cdk5 and Cdk5 protein levels in the contralateral or ipsilateral PL, PL or CG1 after CFA injection (*P<0.05, one-way ANOVA with Newman–Keuls post tests). The data are presented as mean±s.e.m.

These results illustrate that the deactivation of contralateral PL excitatory neurons triggers anxiety-like behaviours and exacerbates heat hyperalgesia in the chronic pain model; however, the underlying mechanism remains unknown. Many protein kinases and ion channels are involved in the regulation of neuronal excitability. For example, Cdk5 is widely expressed in the nervous system and is involved in numerous physiological and pathological processes^20^,^21^. It has been reported that activation of Cdk5 in the peripheral nervous system promotes primary afferent nociceptive signalling^22^,^23^ and influences synaptic transmission in the central nervous system^24^. Stress exposure increases anxiety-like behaviours and Cdk5 activation in the basolateral amygdala^25^. The inhibition of septal Cdk5 blocks enhanced anxiety induced by prior stress^26^. To test whether Cdk5 is involved in the modulation of pain sensation and pain-related anxiety-like behaviours, we first examined Cdk5 and p-Cdk5 protein levels in the contralateral and ipsilateral PL, CG1 or IL 1 or 7 days after CFA injection. These results indicate that Cdk5 protein levels were not altered after CFA injection in any these three regions (Fig. 6a,b), whereas p-Cdk5 levels in the contralateral PL were increased for at least 7 days (Fig. 6a). No significant changes in p-Cdk5 levels were identified in the contralateral CG1 or IL or the ipsilateral PL, CG1 or IL (Fig. 6a,b), suggesting Cdk5 is specifically activated in the contralateral PL after CFA injection.

### Knockdown of Cdk5 reverts the deactivation of PL

**Figure 7:**
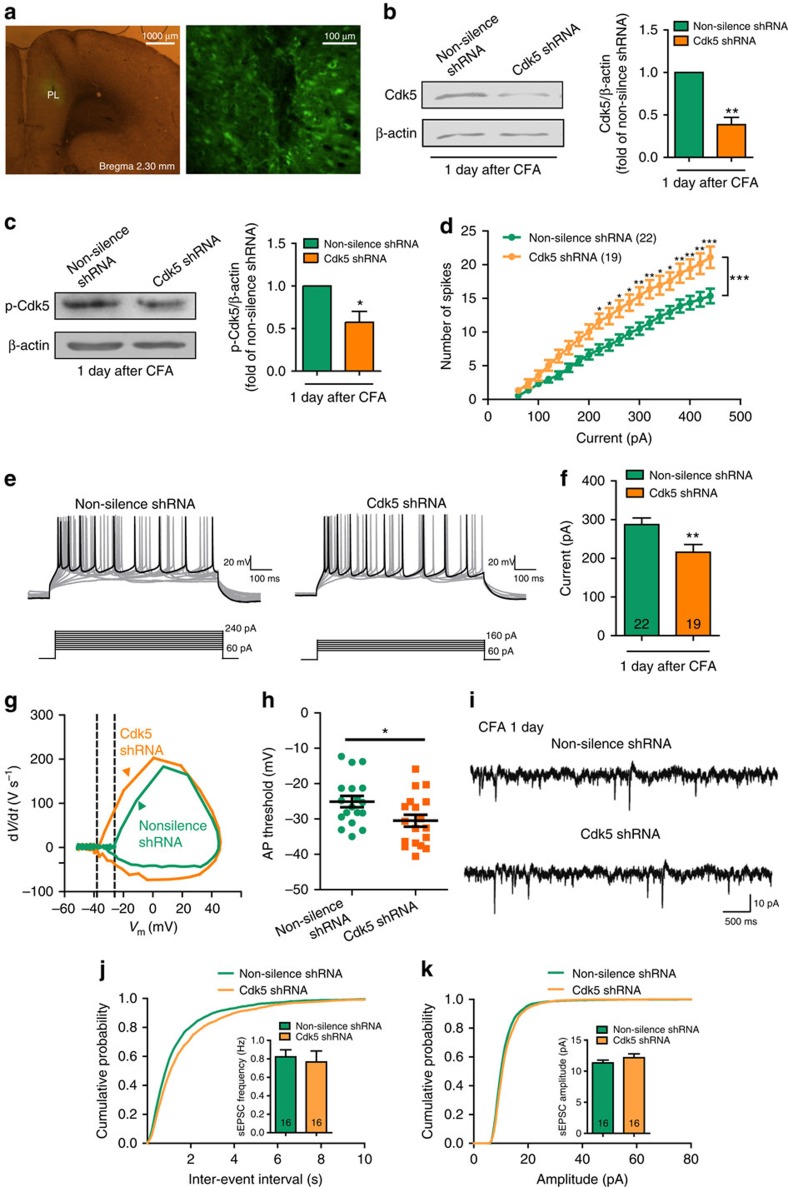
Knockdown of Cdk5 in the contralateral PL reverts the deactivation of PL excitatory neurons. (**a**) Expression of Cdk5 shRNA lentiviruses in the PL (Bregma 2.30 mm). (**b**) Cdk5 protein level after infection of lentiviruses that express Cdk5 shRNA (**P<0.05, paired *t*-test). (**c**) p-Cdk5 protein level after infection of lentiviruses that express Cdk5 shRNA (*P<0.05, paired *t*-test). (**d**) Number of spikes induced by injected currents in the contralateral PL excitatory neurons from the rats injected with lentiviruses that express non-silence shRNA or Cdk5 shRNA 1 day after CFA (*n*=22, 19 neurons, *P<0.05, **P<0.01, ***P<0.001, two-way ANOVA with Bonferroni post tests). (**e**) Examples of AP responses to positive current steps recorded from pyramidal neurons from the non-silence shRNA and Cdk5 shRNA rats 1 day after CFA. (**f**) The current required to drive APs at a frequency of 12.5 Hz was smaller in the Cdk5 shRNA rats (215.8±19.92 pA, *n*=19 neurons) compared with the non-silence shRNA rats (287.3±17.13 pA, *n*=22 neurons; **P<0.01, two-tailed *t*-test). (**g**) Phase plots of APs from the non-silence shRNA and Cdk5 shRNA rats 1 day after CFA evoked by brief current injections. Note the different initial point of the firing threshold. (**h**) Plot diagram summary of the AP thresholds from the non-silence shRNA rats (−25.09±1.590 mV, *n*=18 neurons) and Cdk5 shRNA rats (−30.49±1.692 mV, *n*=18 neurons, *P<0.05, two-tailed *t*-test) 1 day after CFA. (**i**) Representative sEPSC recording traces from the rats injected with lentiviruses that express non-silence shRNA or Cdk5 shRNA. Scale bars, 10 pA and 500 ms. (**j**, **k**) Cumulative probabilities (Frequency distribution, ***P<0.001; Amplitude distribution, **P<0.01; Kolmogorov–Smirnov two sample test) and average sEPSC frequencies and amplitudes from the non-silence shRNA group (frequency: 0.8224±0.7596 Hz, amplitude: 11.32±0.4476, pA, *n*=16 neurons) and the Cdk5 shRNA group (frequency: 0.7664±0.1192 Hz, amplitude: 12.16±0.6138, pA, *n*=16 neurons, two-tailed *t*-test). The data are presented as mean±s.e.m.

To further test whether Cdk5 is involved in the deactivation of contralateral PL excitatory neurons, we expressed an effective short hairpin RNA (shRNA) to Cdk5 in the contralateral PL prior to CFA treatment. One week after virus micro-infusion, CFA was injected in the left hind paw contralateral to the lentivirus (LV)-injected side. The LV that expressed the Cdk5 shRNA infected the PL and decreased both Cdk5 and p-Cdk5 protein levels (Fig. 7a–c).

We next examined the physiology of neurons following knockdown of Cdk5 by calculating the number of APs evoked by current steps, as described above. The decreased number of APs caused by CFA injection was rescued by micro-infusion of Cdk5 shRNA into the PL (Fig. 7d). The input–output curves obtained from neurons in Cdk5 knockdown rats exhibited a leftward shift compared with non-silence shRNA controls. This input–output curve indicated that neurons from the Cdk5 shRNA rats elicited more APs than neurons from non-silence shRNA rats 1 day after CFA injection. The current required to drive spike firing at 12.5 Hz was substantially smaller in the Cdk5 shRNA rats compared with the non-silence shRNA rats 1 day after CFA (Fig. 7e,f). The neurons from Cdk5 shRNA rats also had a less depolarized firing threshold compared with those from naive rats (Fig. 7g,h). The average frequencies and the amplitudes of sEPSCs from both groups were not significantly different, although we detected a slight difference in the cumulative distributions (Fig. 7i–k), suggesting that synaptic transmission was intact. In addition, there were no significant differences in resting membrane potential or input resistance of neurons from the two groups (Supplementary Figs 3c,d). These results demonstrate that Cdk5 knockdown reverses the decreased intrinsic excitability of layer 2/3 pyramidal neurons in the contralateral PL 1 day after CFA, which supports our hypothesis that the deactivation of contralateral PL excitatory neurons 1 day after CFA is a result of the activation of Cdk5 signalling pathway.

### Knockdown of Cdk5 attenuates CFA-induced pain and anxiety

**Figure 8:**
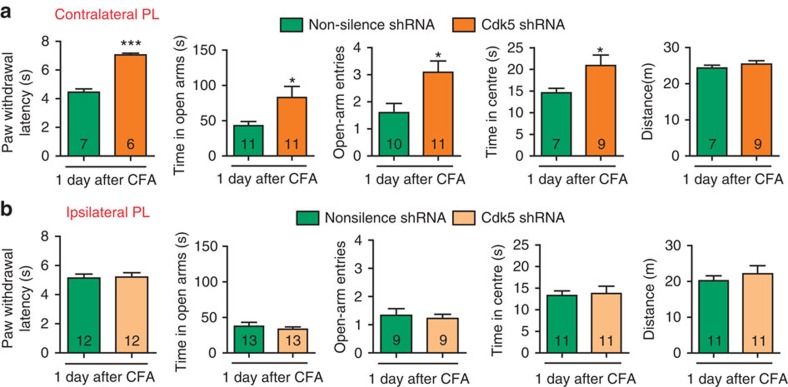
Knockdown of Cdk5 in the contralateral PL attenuates CFA-induced behavioural impairments. (**a**, **b**) Paw withdrawal latency, elevated plus maze and open field test after contralateral or ipsilateral micro-infusion of lentiviruses that express the Cdk5 shRNA lentivirus or non-silence shRNA (*P<0.05, ***P<0.001, two-tailed *t*-test). The data are presented as mean±s.e.m.

To directly test whether Cdk5 is responsible for CFA-induced behavioural impairments, we measured the PWL and tested the EPM and OFT after a contralateral or an ipsilateral Cdk5 shRNA infection. Consistent with the above results, following Cdk5 knockdown in the contralateral PL, the PWL, the time in the open arms, open-arm entries and the time in centre were augmented (Fig. 8a), whereas the ipsilateral PL did not affect these behaviours (Fig. 8b).

These results confirm our hypothesis that the deactivation of the contralateral PL excitatory neurons by Cdk5 activation is responsible for the initiation of pain-induced anxiety-like behaviours and the modulation of pain sensation.

## Discussion

In the present study, we demonstrate that the intrinsic excitability of excitatory pyramidal neurons in layers 2/3 of contralateral PL is decreased 1 day after CFA injection to the hind paw, whereas synaptic transmission is enhanced. Optogenetic activation of contralateral PL excitatory neurons in CFA mice exerts analgesic and anxiolytic effects, whereas optogenetic inhibition of PL excitatory neurons induces anxiety-like behaviours without affecting pain sensation. Cdk5 is activated in the contralateral PL and Cdk5 knockdown in the contralateral PL by Cdk5 shRNA reverses the deactivation of contralateral PL excitatory neurons as well as the behavioural impairments induced by CFA. One potential interpretation based on our study is that in chronic pain states, the deactivation of the contralateral PL excitatory neurons by Cdk5 activation promotes CFA-induced anxiety-like behaviours and heat hyperalgesia, which indicates that the mPFC is a key node of the circuitry involved in both pain and anxiety processing.

Chronic pain is thought to be caused by aberrant neuronal responses along the pain transmission pathway from the dorsal root ganglion to the spinal cord, thalamus and cortex^27^. The sensitization of peripheral nociceptors leads to primary hyperalgesia and pain. However, the functional and morphological changes in the central nervous system significantly contribute to the maintenance of chronic pain and its comorbidities. In the present study, we demonstrate that the PL but not the CG1 or IL of the mPFC is involved in inflammatory heat hyperalgesia and anxiety-like behaviours, which indicates that the three main sub-divisions of the mPFC present different roles in pain sensation and affective pain. There are intrinsic connections between the PL and IL^28^ and previous studies of oscillatory neural activity in these regions suggest their interaction^29^,^30^, contributing to the complex functions of the mPFC. To identify a role for PL excitatory neurons, we both bilaterally damaged and optogenetically activated the IL and repeated the behavioural tests and showed that these manipulations had no effects on anxiety-like behaviours and pain sensation. Our results are consistent with a recent study demonstrating that activation of the PL-nucleus accumbens (NAc) pathway in a spared nerve injury model produces antinociceptive effects and reduces the affective symptoms of pain^31^. However, we cannot fully exclude the role of the IL because we did not test the role of IL excitatory neurons in naive mice.

Pain signalling is conveyed to the contralateral cortex from the spinal cord. In our study, only lesions of the contralateral, not ipsilateral PL attenuated CFA-induced anxiety-like behaviours and heat hyperalgesia, supporting our hypothesis that the contralateral mPFC controls initiation of anxiety-like behaviours modulation of chronic pain. Further electrophysiological studies indicated that the intrinsic excitability of the contralateral PL excitatory neurons decreased 1 day after CFA. Optogenetic activation of contralateral PL excitatory neurons exerted analgesic and anxiolytic effects, which were not observed following activation of ipsilateral PL excitatory neurons. These results suggest that pain signalling is transmitted to the contralateral PL, where it results in decreased intrinsic excitability, with the ipsilateral side largely unaffected. However, the activation of either contralateral or ipsilateral PL excitatory neurons increased travel distance in the OFT, which suggests a general role for the PL in locomotor activity.

To gain insight into the neurobiological mechanisms of high-order sensation and cognitive functions, it is important to understand the critical transformational properties of information input and output in the mPFC, as well as the micro-circuitry formed in the mPFC. In this study, we focused on layer 2/3 pyramidal neurons, which are directly associated with pain sensation and pain-related behaviours^32^,^33^,^34^. We demonstrated that layer 2/3 pyramidal neurons of the PL are critical for integration and transmission in the pathological process of inflammatory pain. The intrinsic excitability of layer 2/3 neurons was decreased, whereas the synaptic transmission was increased 1 day after CFA injection. We speculate that when pain occurs, pain-related information from peripheral nociceptors is continuously transmitted to the PL, which leads to an increased synaptic transmission. However, as pain persists and becomes chronic, the intrinsic excitability of layer 2/3 pyramidal neurons in the PL decreases because of the continuous activation by inflammatory stimuli. Neurons with decreased intrinsic excitability fail to generate APs, which indicates that the integration and output of these neurons is affected. This dysfunction of layer 2/3 neurons in PL blocks the processing and transmission of the pain signalling, which in turn, initiates anxiety-like behaviours and exacerbates chronic pain. The regulation of pain in layer 2/3 pyramidal neurons of the PL interprets one potential mechanism of pain and pain-related anxiety in the central nervous system. Here, for the first time, we demonstrated the relationship of the deactivation of contralateral PL excitatory neurons, heat hyperalgesia and anxiety-like behaviours.

Moreover, our work identifies the activation of the Cdk5 signalling pathway as a potential mechanism underlying the deactivation of contralateral PL excitatory neurons, which suggests that Cdk5 acts as a key negative regulator of neuronal excitability in the PL. This finding is consistent with a previous study that neurons in the NAc from CaMKII-Cre Cdk5 conditioned knockout mice exhibit increased intrinsic excitability^35^. However, how Cdk5 negatively regulates intrinsic excitability remains unknown. The intrinsic excitability of neurons is driven by dynamic modulation of ion channel expression, localization and/or function^36^, which can be mediated by the direct or indirect phosphorylation of Cdk5. A previous study has shown that mGluR1 and GABA_A_ receptors are involved in the deactivation of medial prefrontal cortical neurons in an arthritis pain model^14^. The potential candidates that act downstream of Cdk5 should also be involved in the firing of APs. Future studies must be performed to elucidate the additional mechanisms by which Cdk5 may negatively regulate the intrinsic excitability of PL excitatory neurons.

There are three issues to be noted: first, both chemical lesion and optogenetic activation of the contralateral PL exerted analgesia and anxiolytic effects. The results from the chemical lesion and optogenetic studies appear contradictive. The mPFC consists of a heterogeneous collection of neurons, including excitatory pyramidal neurons and local GABAergic interneurons^37^. In a chemical lesion, all PL neurons, including excitatory and inhibitory neurons, are eliminated, whereas the optogenetics manipulation only activated excitatory neurons. Evidence suggests that GABAergic interneurons are involved in the modulation of excitatory neuron activity in the PL^38^,^39^. This contradiction suggests that inhibitory neurons may also play a role in the modulation of anxiety-like behaviours and pain sensation. The role of GABAergic interneurons in chronic pain needs to be further clarified. Second, optogenetic activation of contralateral PL excitatory neurons in CFA mice not only increased the time in the centre but also the travel distance, which raises the question of whether the relief of anxiety-like behaviours is a result of the increased travel distance. The increased distance is consistent with previous work showing that inhibition of GABA synthesis in the PFC increases locomotor activity during a 24-h testing period^40^. It has been reported that the PFC has connections with motor structures, enabling a direct translation of PFC function outcome into behaviour^41^ and may be the reason why the distance is increased with the activation of contralateral PL excitatory neurons. Our experiments further demonstrated that optogenetic activation of ipsilateral PL excitatory neurons in CFA mice only increased the travel distance without alleviating the anxiety-like behaviours. These results rules out the possibility that the relief of anxiety-like behaviours is because of the increased travel distance. Third, optogenetic inhibition of PL excitatory neurons did not influence the pain sensation. Previous studies have suggested that the PFC is involved in thalamic-PAG-dorsal horn top-down modulation of pain^42^. We speculate that the activation of PL excitatory neurons strongly activates the descending inhibitory pathway, which thus increases the pain threshold. This would account for the finding that optogenetic inhibition of PL excitatory neurons did not influence pain sensation.

Nevertheless, our findings provide novel insights into the role of contralateral PL excitatory neurons in the regulation of pain sensation and affective pain. Cdk5 signalling is one potential mechanism responsible for the PL changes. Furthering our understanding of the underlying mechanisms of chronic pain may offer better therapeutic methods that target multiple aspects of pain with one treatment.

## Methods

### Experimental animals

Male Sprague-Dawley rats (200–250 g) and male C57BL/6J (20–25 g) mice were used, which were supplied by the Animal Center of Peking University, Health Science Center and the Guangdong Medical Laboratory Animal Center, respectively. The animals were housed in a constant temperature at 23±1 °C under a 12 h light/dark cycle. Food and water were available *ad libitum*. The animals were acclimated for at least 5 days before the beginning of the experimental procedures. All experimental procedures comply with the guidelines of the Animal Care and Use Committee of Peking University. CFA (100 μl for rats, 10 μl for mice, Sigma-Aldrich, St Louis, MO, USA) was injected into the plantar surface of the hind paw, and the animals without CFA injection comprised the naive group.

### Assessment of thermal hyperalgesia

Thermal hyperalgesia was evaluated by following a thermal stimulus paradigm adapted from published reports of ref. 43. Before testing, the animals were allowed to acclimatize to the environment for 20 min. The radiant heat source was adjusted to a range of 12–15 s for rats and 5–10 s for mice as the baseline latencies with a cutoff time of 30 s to prevent tissue damage. To record the PWL in response to the heat stimulus, each hind paw was measured four times with a 5-min interval, and the mean value was recorded. The experimental procedures were performed in a double-blind manner.

### Elevated plus maze

The maze consisted of four equally illuminated plastic arms (rats, 12 × 50 cm; mice, 5 × 35 cm) that radiated at square angles from a central platform (rats, 12 × 12 cm; mice, 5 × 5 cm) 50 cm above the floor. The maze was placed in a silent and dimly lit room with an illumination of 100–200 lux. Two opposite arms were enclosed by 40 cm high plastic walls. The other two arms were open. The animals were released from the central platform, with their face pointing toward an enclosed arm. Animal behaviour was recorded with a camera. The time spent in the open arms and open-arm entries were measured using ANY-maze (Stoelting Co., IL, USA).

### Open field test

The apparatus consisted of a large area composed of plastic, surrounded by walls that were 100 cm high. The floor was 100 × 100 cm for rats and 50 × 50 cm for mice^44^; the overall illumination was 100–200 lux. Each animal was gently placed in the centre of the open field, and its behaviour was videotaped. The time in the centre (rats, 60 × 60 cm; mice, 25 × 25 cm) and the distance the animals travelled were measured using ANY-maze (Stoelting Co., IL, USA).

### Lesions of the PL, CG1 and IL

The rats were anesthetized with 10% chloral hydrate (0.4 g kg^−1^, i.p.) and positioned in a stereotaxic instrument (RWD, Shenzhen, China). A 5-μl Hamilton microsyringe was unilaterally placed in the mPFC sub-regions according to the atlas of Paxinos and Watson (1997) (CG1: AP, +2.8, ML, ±0.5, DV, −2.6; PL: AP, +2.8, ML, ±0.5, DV, −4; IL: AP, +2.8, ML, ±0.5, DV, −5 mm). For each side of the PL, CG1 and IL, a 0.5 μl volume of a 0.1-M solution of QA in phosphate buffered saline (0.1 mol l^−1^, pH=7.4) was administered in 1 min (0.5 μl min^−1^). All injections were followed by an additional 2 min to allow diffusion before removal of the injection needle. After the surgery, the animal was returned to its home cage. One week after the surgery, anxiety-like behaviours and PWL were assessed.

### Brain slice preparation

Male Sprague-Dawley rats (160–180 g) were used for the experiments that involved whole-cell patch clamp recordings. The rats were anesthetized with pentobarbital sodium (40 mg kg^−1^, i.p.) (Merck, Darmstadt, Germany). The brains were quickly removed (within 1 min) and submerged in ice-cold artificial cerebrospinal fluid (ACSF) that contained (in mM) 119 NaCl, 2.5 KCl, 1 CaCl_2_, 3 MgSO_4_, 1 NaH_2_PO_4_, 26.2 NaHCO_3_ and 16 glucose (saturated with 95% O_2_–5% CO_2_). Coronal bilateral slices (400 μm in thickness) that contained the mPFC were then cut on a vibroslice (1,000+, Pelco 102, Ted Pella, CA, USA). The anatomic locations of these slices were assessed according to the atlas of Paxinos and Watson (1997) and encompassed the region equivalent to AP: +2.7 to +3.5 mm, ML: 0.0–1.2 mm, and DV: −3.0 to −4.2 mm. The slices were incubated in an oxygenated (95% O_2_–5% CO_2_) ACSF incubation bath and incubated at room temperature for at least 1 h before a single slice was transferred to a submerged recording chamber. The chamber was perfused with a pump (Peri-Star 291, World Precision Instruments, FL, USA) at a rate of 2–3 ml min^−1^ with ACSF that consisted of (in mM): 119 NaCl, 2.5 KCl, 2.5 CaCl_2_, 1.3 MgSO_4_, 1 NaH_2_PO_4_, 26.2 NaHCO_3_ and 16 glucose (saturated with 95% O_2_–5% CO_2_). All experiments were performed at room temperature (∼23 °C).

### Whole-cell patch clamp recordings

A slice was viewed with an upright microscope (Axioskop Fsmot, Zeiss, Germany) equipped with infrared-differential interference contrast optics. Layer 2/3 pyramidal cells of the contralateral PL were easily recognizable via a × 40 water-immersion lens. The recording pipettes (3–5 MΩ) were filled with solution that contained (in mM): 140K-gluconate, 8 NaCl, 2 MgCl_2_, 1 EGTA, 10 HEPES, 2 Mg-ATP and 0.3 Na-GTP (adjusted to pH 7.2 with KOH, 290–320 mOsm).

Voltage and current signals were recorded from layer 2/3 neurons using an Axon 200B amplifier (Axon Instruments, CA). The spike firing pattern of the AP was used to electrophysiologically identify pyramidal neurons, which exhibited significant spike frequency adaptation in response to a depolarization current. The APs were recorded using the current-clamp mode. For sEPSC recordings, picrotoxin (50 μM) was added to block GABA_A_ receptor-mediated inhibitory synaptic currents. The sEPSCs were recorded using the voltage-clamp mode and neurons were voltage clamped at −70 mV. The series resistance (Rs) was monitored at regular intervals throughout the recording experiment. Cells with Rs larger than 25 MΩ during the recordings were discarded. Data were acquired and analysed with the Axoclamp 200B amplifier and pCLAMP software (Axon Instruments, Foster City, CA, USA). The analysis of sEPSCs was performed blind using MiniAnalysis software (Synaptosoft). The average amplitude and frequency of sEPSCs was determined by the two-tailed *t*-tests. 50 random points selected from each cell were concatenated to describe the cumulative distributions of sEPSC in each condition and then compared by a Kolmogorov–Smirnov two sample test. *P*<0.05 was considered to be statistically significant. Data are presented as mean±s.e.m.

### Optogenetics and behavioural tests

Male C57BL/6J (20–25 g) mice were used in the optogenetic experiments. Virus encoding AAV5-CaMKIIα-ChR2-mCherry, AAV5-CaMKIIα-NpHR3.0-mCherry and AAV5-CaMKIIα-mCherry was a generous gift from Liping Wang’s lab.

The mice were anesthetized with 0.5% pentobarbital sodium (80 mg kg^−1^, i.p.) and positioned in a stereotaxic frame. After craniotomy, the mice were injected with AAV5-CaMKIIα-ChR2-mCherry, AAV5-CaMKIIα-NpHR3.0-mCherry or AAV5-CaMKIIα-mCherry into the PL (AP, +1.9, ML, −0.3, DV, −2.3 mm), the CG1 (AP, +1.9, ML, −0.3, DV, −2.0 mm) or the IL (AP, +1.7, ML, −0.3, DV, −2.6 mm). A volume of 0.6 μl virus was delivered at a rate of 0.1 μl min^−1^ with a 10 μl microsyringe with a 33 gauge metal needle (Neuros; Hamilton, Reno, NV, USA), which was controlled by a microsyringe pump (UMP3; WPI, Sarasota, FL, USA) and its controller (Micro4; WPI, Sarasota, FL, USA). After the injection, the needle was maintained in the place for an additional 5 min to facilitate the diffusion of the virus and then slowly withdrawn. Then, an optical fibre was immediately implanted (NA=0.37, Φ=200 μm; Fiblaser, Shanghai, China) 0.3 mm above the injection site. The optical fibers were first fixed to the skull with a layer of cyanoacrylate glue (Permabond, Englewood, FL, USA) followed by dental cement (New Century, Shanghai, China).

Multi-channel recordings and behavioural tests were performed at least 4 weeks later to enable viral expression. In behavioural tests, the mice were placed in the centre of the plus maze or open field and allowed to freely explore for 3 min; light was subsequently delivered for 3 min and was shut-off for the next 3 min. The PWL was measured every 3 min for four times before light stimulation, with light stimulation and after light stimulation, respectively. The mean value was calculated. For the activation of the excitatory neurons, 20 Hz, 6–9 mW 473 nm blue light was delivered by a blue light laser (MBL-473/200 mW; Fiblaser, Shanghai, China), while for the inhibition of the excitatory neurons, continuous 6–9 mW 593 nm yellow light was delivered by a yellow light laser (MBL-593/200 mW; Fiblaser, Shanghai, China).

### Multi-channel recordings

The optrode was an optic fibre attached with four surrounding tetrodes. The fibre tip was 0.5 mm shorter than the tetrode tips. The input end of the fibre was embedded into a ceramic ferrule, which could be connected to the laser source via an FC/PC connector. The tips of all tetrodes (12.7 μm nickel-chromium wires, CFW, CA, USA) were plated with platinum (chloroplatinic acid solution) to a final impedance of 0.5–0.6 MΩ. The mice were anesthetized with 0.5% pentobarbital sodium and positioned in a stereotaxic frame. The multi-channel optrode was injected in the PL (AP, +1.9, ML, −0.3, DV, −2.3 mm). The baseline firing of the PL neurons was recorded by an OmniPlex D Neural Data Acquisition System for 1 min, followed by six rounds of 30 s light stimulation and 30 s light off. Continuous wide-band data were imported into Plexon Offline Sorter software (Plexon, Inc., Dallas, TX, USA) for the spike detection and offline sorting.

### Western blotting

The rats were anesthetized with 10% chloral hydrate (0.4 g kg^−1^, i.p.) and the brains were quickly removed and frozen in N-hexane −70 °C for ∼40 s. Bilateral tissue punches of the PL, IL and CG1 were obtained from 60-μm thick sections taken on a sliding freezing microtome^45^. Western blotting experiments were performed according to the protocols previously described^23^. In brief, tissues were homogenized in ice-cold lysis buffer (50 mM Tris, pH 7.4, 150 mM NaCl, 5 mM EGTA, 0.5% NP-40, 10 mM NaF, and 1 mM PMSF) and rotated at 4 °C for 1 h before the supernatant was extracted. The supernatant was extracted by centrifugation at 12,000*g* at 4 °C for 5 min. Equal amounts of protein extracts were denatured and subjected to SDS–polyacrylamide gel electrophoresis. After separation, proteins were transferred to nitrocellulose membranes (Bio-Rad). The membranes were blocked with 5% nonfat milk in TBST (25 mM Tris, pH 7.4, 137 mM NaCl, 2.7 mM KCl, and 0.05% Tween 20) for 1 h at room temperature and incubated with goat monoclonal anti-p-Cdk5 (1:100 dilution, Sc-12918, Santa Cruz Biotechnology, Dallas, TX, USA), monoclonal anti-Cdk5 antibody (1:2,000 dilution, Upstate Biotechnology, Buffalo, NY, USA) or monoclonal anti-β-actin antibody (1:2,000 dilution, Sigma-Aldrich, St Louis, MO, USA) overnight at 4 °C. After washing three times with TBST, the membranes were incubated with secondary antibody (1:2,000 dilution) overnight at 4 °C, then washed again, and finally developed with ECL solutions (Santa Cruz Biotechnology, Dallas, TX, USA).

### Micro-infusion PL of Cdk5 ShRNA

Surgery was performed according to the protocol described above. ShRNA LVs that co-expressed EGFP and shRNAs against rat Cdk5 were purchased from Shanghai GeneChem Co., Ltd. The target mRNA sequence was as follows: CCAAACUGCCAGACUAUAA. LVs that expressed shRNAs that targeted a non-specific sequence (TTCTCCGAACGTGTCACGT) were used as the controls. One week after the surgery, whole-cell patch recordings were performed and behaviours were assessed.

### Statistical analysis

The data are presented as means±s.e.m. Comparisons between two groups were performed using Student’s unpaired two-tailed *t*-test or paired *t*-test. Comparisons between three groups were performed using one-way ANOVA with Newman–Keuls post tests. Comparisons between two groups with different time points were performed using two-way ANOVA with Bonferroni post tests. The criterion for statistical significance was *P*<0.05, and differences were calculated using GraphPad Prism 5.0.

## Additional information

**How to cite this article:** Wang, G.-Q. *et al*. Deactivation of excitatory neurons in the prelimbic cortex via Cdk5 promotes pain sensation and anxiety. *Nat. Commun.* 6:7660 doi: 10.1038/ncomms8660 (2015).

## Supplementary Material

Supplementary Information Supplementary Figures 1-9
