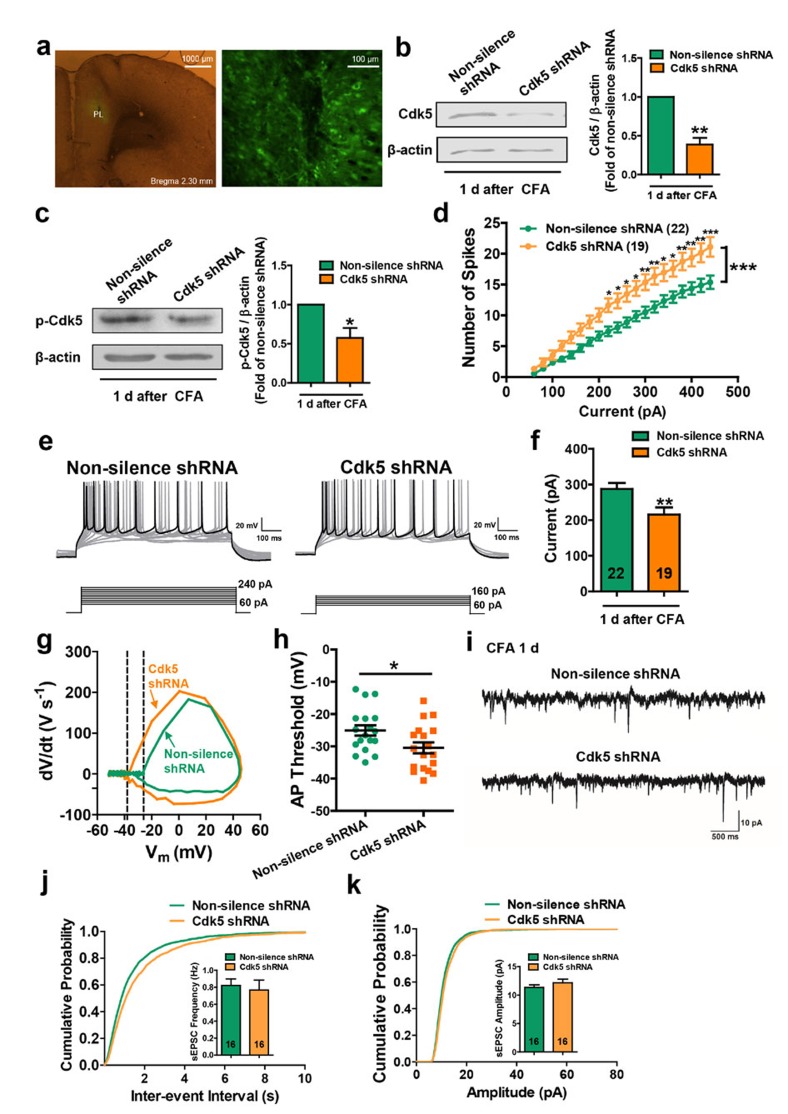# Corrigendum: Deactivation of excitatory neurons in the prelimbic cortex via Cdk5 promotes pain sensation and anxiety

**DOI:** 10.1038/ncomms12705

**Published:** 2016-10-10

**Authors:** Guo-Qiang Wang, Cheng Cen, Chong Li, Shuai Cao, Ning Wang, Zheng Zhou, Xue-Mei Liu, Yu Xu, Na-Xi Tian, Ying Zhang, Jun Wang, Li-Ping Wang, Yun Wang

Nature Communications 6: Article number: 766010.1038/ncomms8660 (2015); Published: 07 16 2015; Updated: 10 10 2016

In Fig. 7i of this Article, a different section of the control trace was used to represent the Cdk5 shRNA trace. The correct version of Fig. 7, including a new representative Cdk5 shRNA trace in panel i, appears below as Fig. 1. The original conclusions are not affected by this change and the raw data files from which these traces were selected are now available as Supplementary Data 1 and 2.

## Supplementary Material

Supplementary Data 1 Non-silence shRNA-primary data

Supplementary Data 2 Cdk5 shRNA-primary data

## Figures and Tables

**Figure 1:**