# Supplementary material for: Deactivation of excitatory neurons in the prelimbic cortex via Cdk5 promotes pain sensation and anxiety
Source: Nat Commun. 2015 Jul 16;6:7660. doi: 10.1038/ncomms8660 (PMC4518290; doi:10.1038/ncomms8660)
Supplement: Supplementary Information — Supplementary Figures 1-9 [file ncomms8660-s1.pdf]

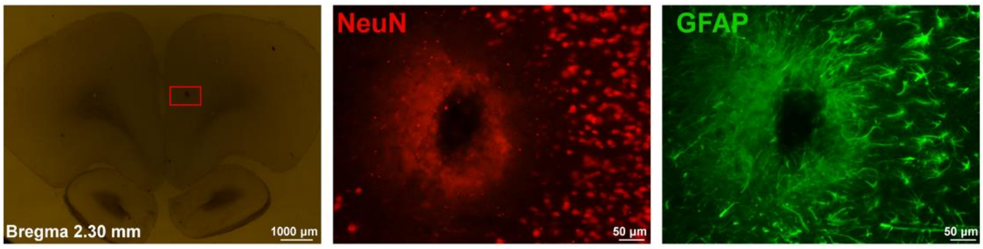

Figure S1. Injection of quinolinic acid into the PL induces the neuronal loss in the PL. The immunostaining of NeuN and GFAP 7 d after the injection of quinolinic acid into the PL. Bregma 2.30 mm. Scale bar, left, 1000 μm; middle and right, 50 μm.

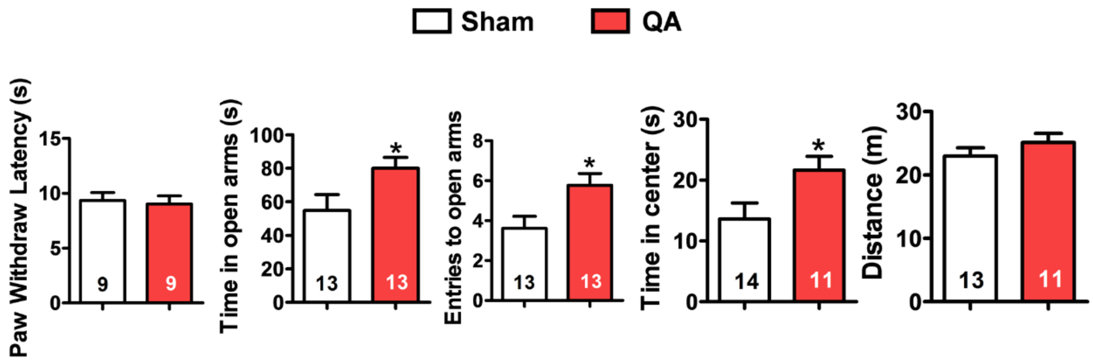

Figure S2. Bilateral lesion of the PL attenuates anxiety-like behaviors without affecting the paw withdrawal latency. Paw withdrawal latency, elevated plus maze, open field test and locomotor activity of the rats after bilateral lesion of the PL by quinolinic acid (*$P<0.05$, two-tailed *t*-test).

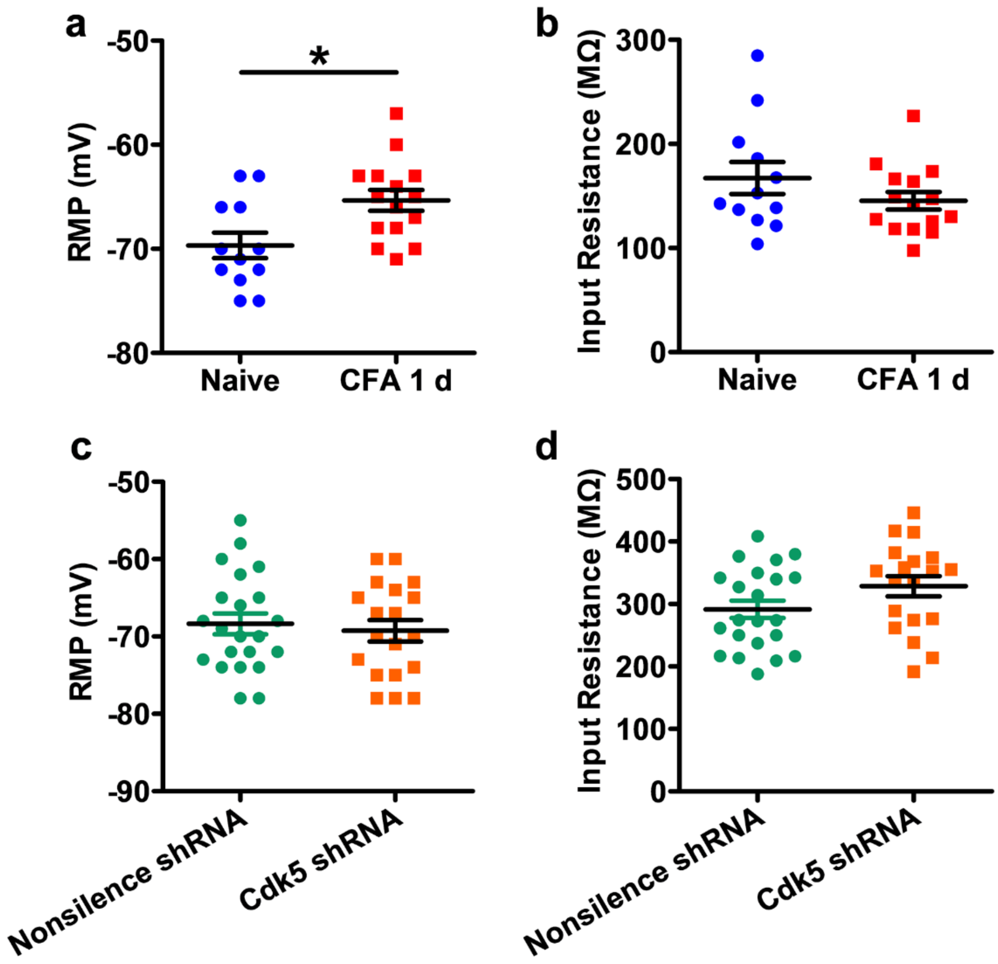

Figure S3. Membrane properties of layer 2/3 pyramidal neurons in the PL.

(a, b) Resting membrane potential and input resistance of neurons from the naïve rats and the rats 1 d after CFA. Neurons from the rats 1 d after CFA exhibited mildly more positive resting membrane potentials compared with the naïve rats (naïve, -69.67 ±1.221 mV, n=12 neurons; CFA 1 d, -65.33 ±0.9984 mV, n=15 neurons; *P<0.05, two-tailed t-test). (c, d) Resting membrane potential and input resistance of neurons from the nonsilence shRNA and Cdk5 shRNA rats 1 d after CFA. No significant differences were identified between the groups (two-tailed t-test).

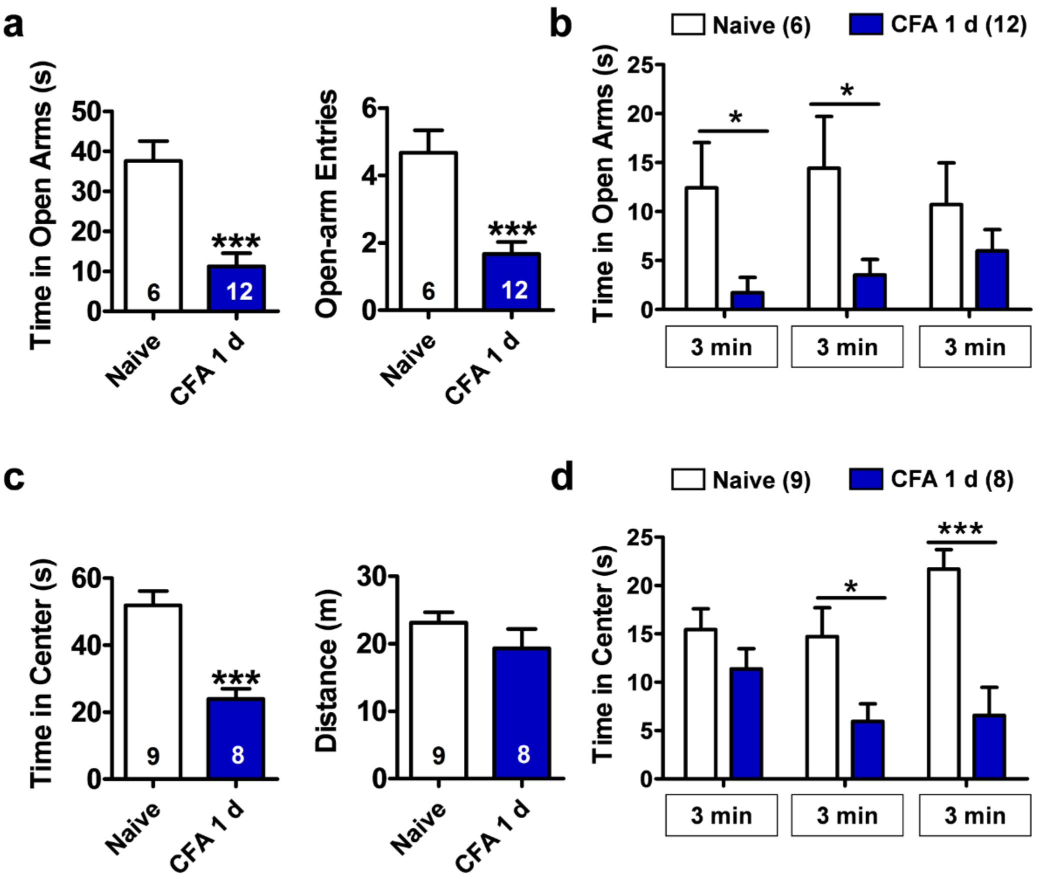

Figure S4. CFA induces the anxiety-like behaviors in C57BL/6J mice. (a) Elevated plus maze of 9-min test time (n=6, 12 animals, ****P*<0.001, two-tailed *t*-test) in the mice 1 d after CFA injection. (b) The time in the open arms of three continuous 3 min in the mice 1 d after CFA injection (n=6, 12 animals, **P*<0.05, two-way ANOVA with Bonferroni post-tests). (c) Open field test of 9-min test time (n=9, 8 animals, ****P*<0.001, two-tailed *t*-test) in the mice 1 d after CFA injection. (d) The time in the center of three continuous 3 min in the mice 1 d after CFA injection (n=9, 8 animals, **P*<0.05, ****P*<0.001, two-way ANOVA with Bonferroni post-tests).

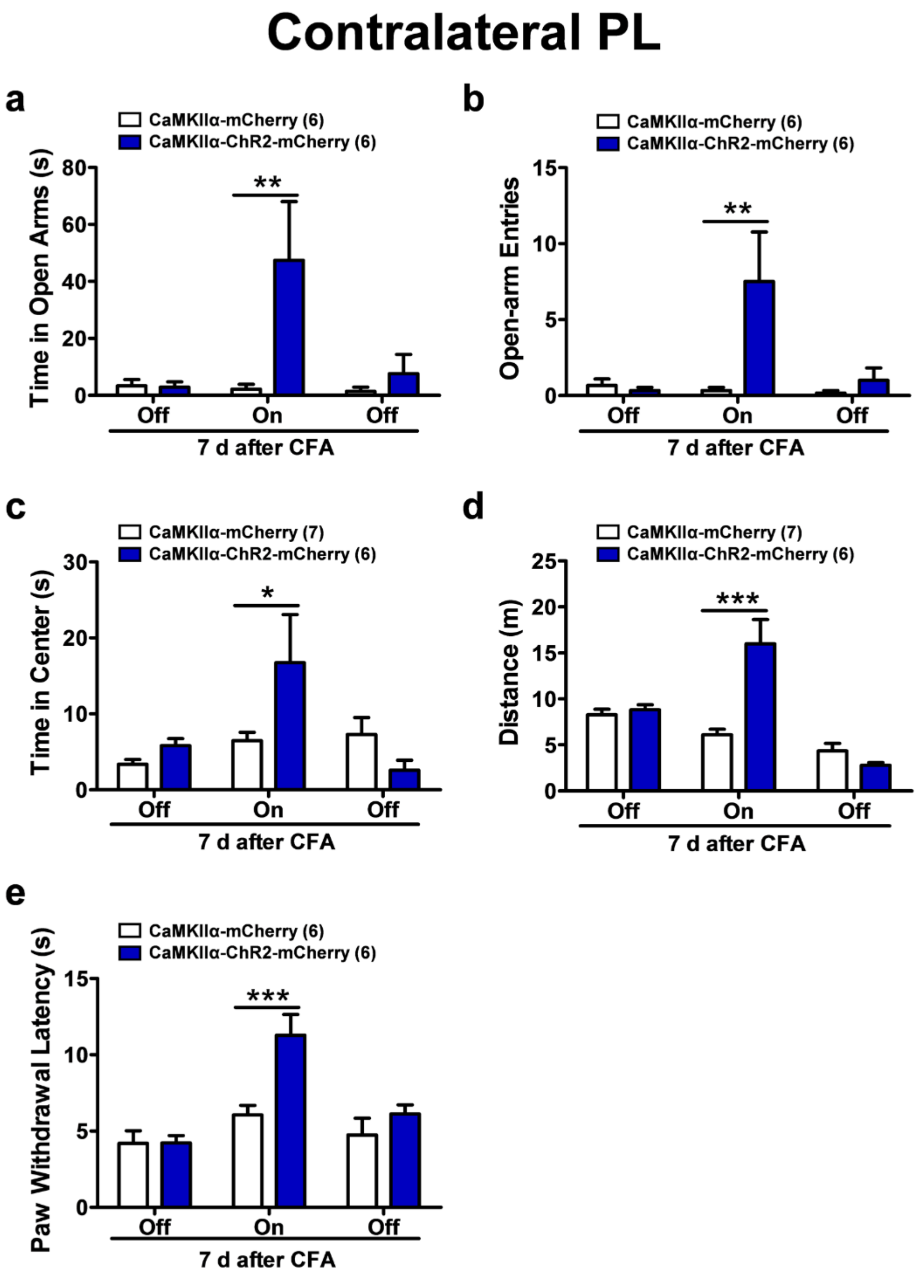

Figure S5. Optogenetic activation of the contralateral PL excitatory neurons 7 d after CFA attenuates CFA-induced heat hyperalgesia and anxiety-like behaviors. Elevated plus maze, open field test, locomotor activity and paw withdrawal latency in the CFA mice of the AAV-CaMKIIα-mCherry or AAV-CaMKIIα-ChR2-mCherry virus injection with 20 Hz, 6–9 mW 473 nm blue light off-on-off stimulation (*P<0.05, **P<0.01, ***P<0.001, two-way ANOVA with Bonferroni post-tests).

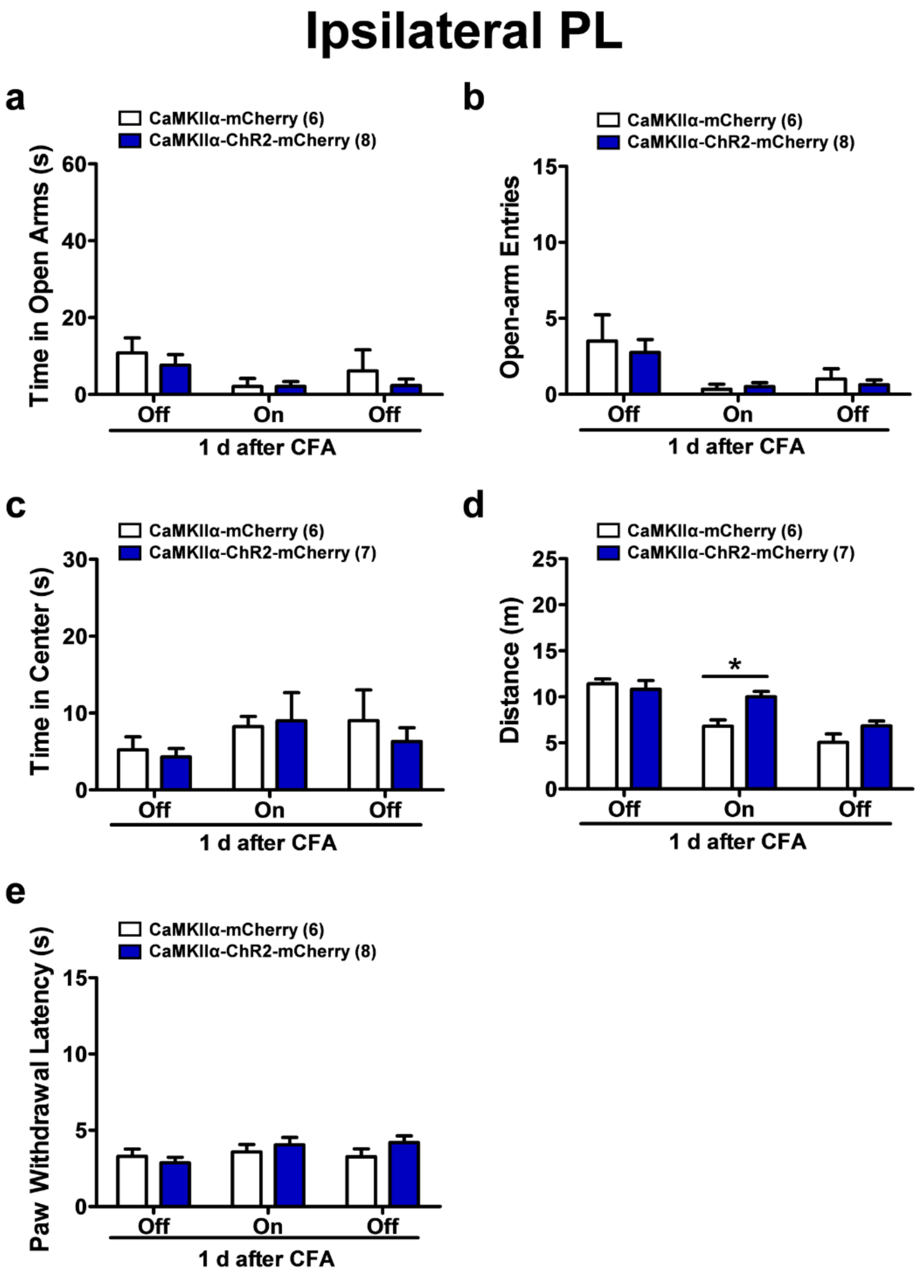

Figure S6. Optogenetic activation of ipsilateral PL excitatory neurons does not reverse CFA-induced heat hyperalgesia and anxiety-like behaviors. Elevated plus maze, open field test, locomotor activity and paw withdrawal latency in the CFA mice of the AAV-CaMKIIα-mCherry or AAV-CaMKIIα-ChR2-mCherry virus injection with 20 Hz, 6–9 mW 473 nm blue light off-on-off stimulation (*$P<0.05$, two-way ANOVA with Bonferroni post-tests).

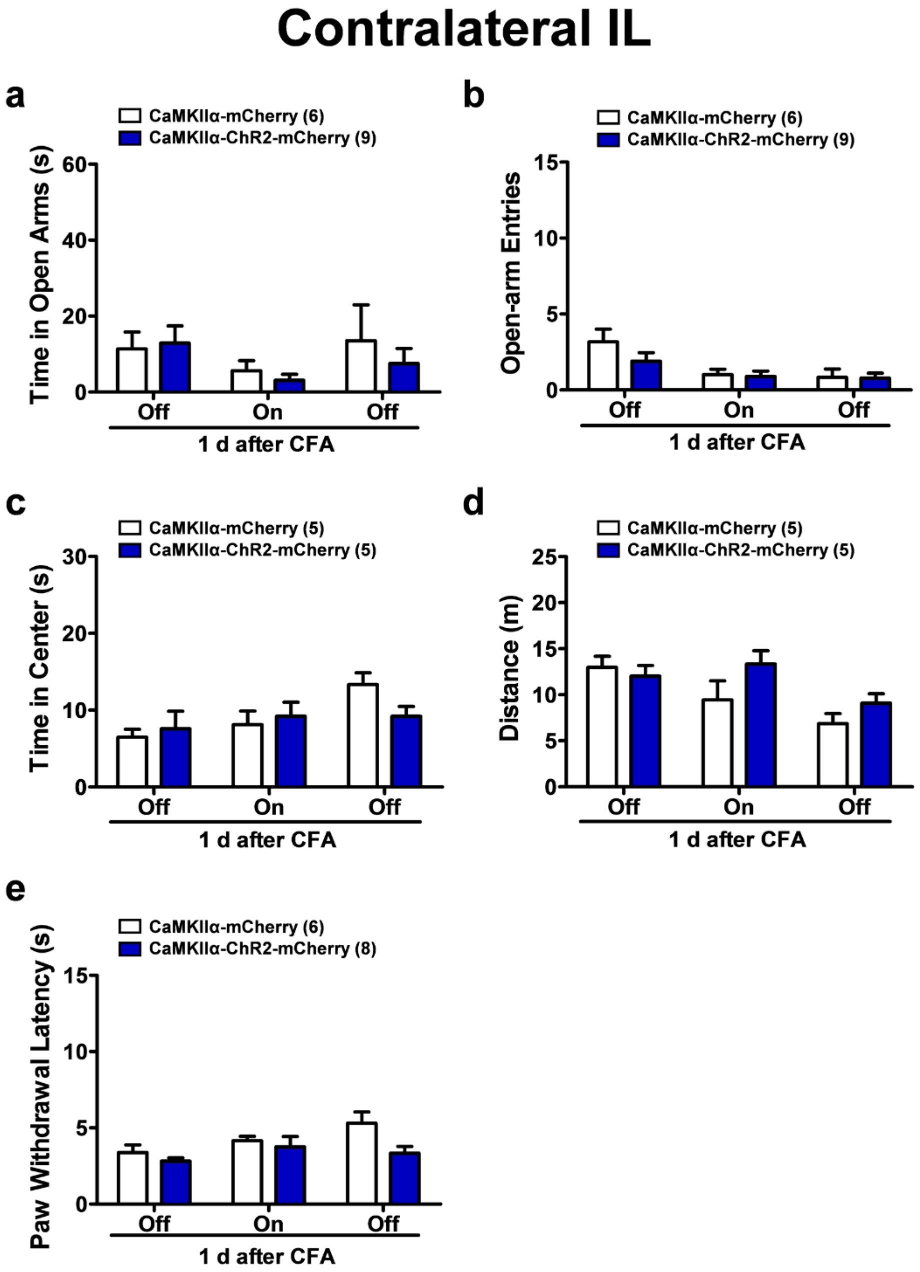

Figure S7. Optogenetic activation of the contralateral IL excitatory neurons does not reverse CFA-induced heat hyperalgesia and anxiety-like behaviors. Elevated plus maze, open field test, locomotor activity and paw withdrawal latency in the CFA mice of the AAV-CaMKIIα-mCherry or AAV-CaMKIIα-ChR2-mCherry virus injection with 20 Hz, 6–9 mW 473 nm blue light off-on-off stimulation (two-way ANOVA with Bonferroni post-tests).

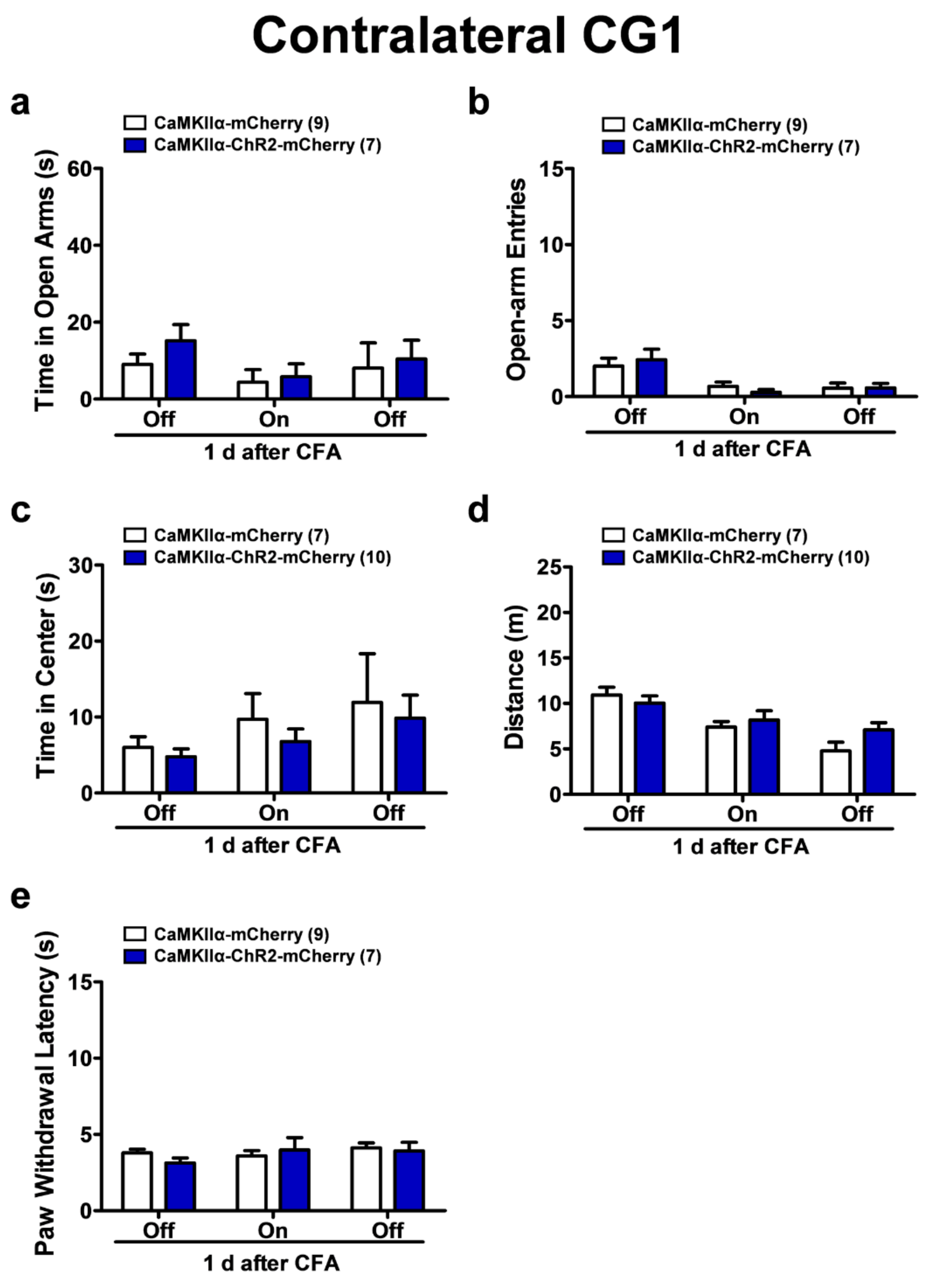

Figure S8. Optogenetic activation of the contralateral CG1 excitatory neurons does not reverse CFA-induced heat hyperalgesia and anxiety-like behaviors. Elevated plus maze, open field test, locomotor activity and paw withdrawal latency in the CFA mice of the AAV-CaMKIIα-mCherry or AAV-CaMKIIα-ChR2-mCherry virus injection with 20 Hz, 6–9 mW 473 nm blue light off-on-off stimulation (two-way ANOVA with Bonferroni post-tests).

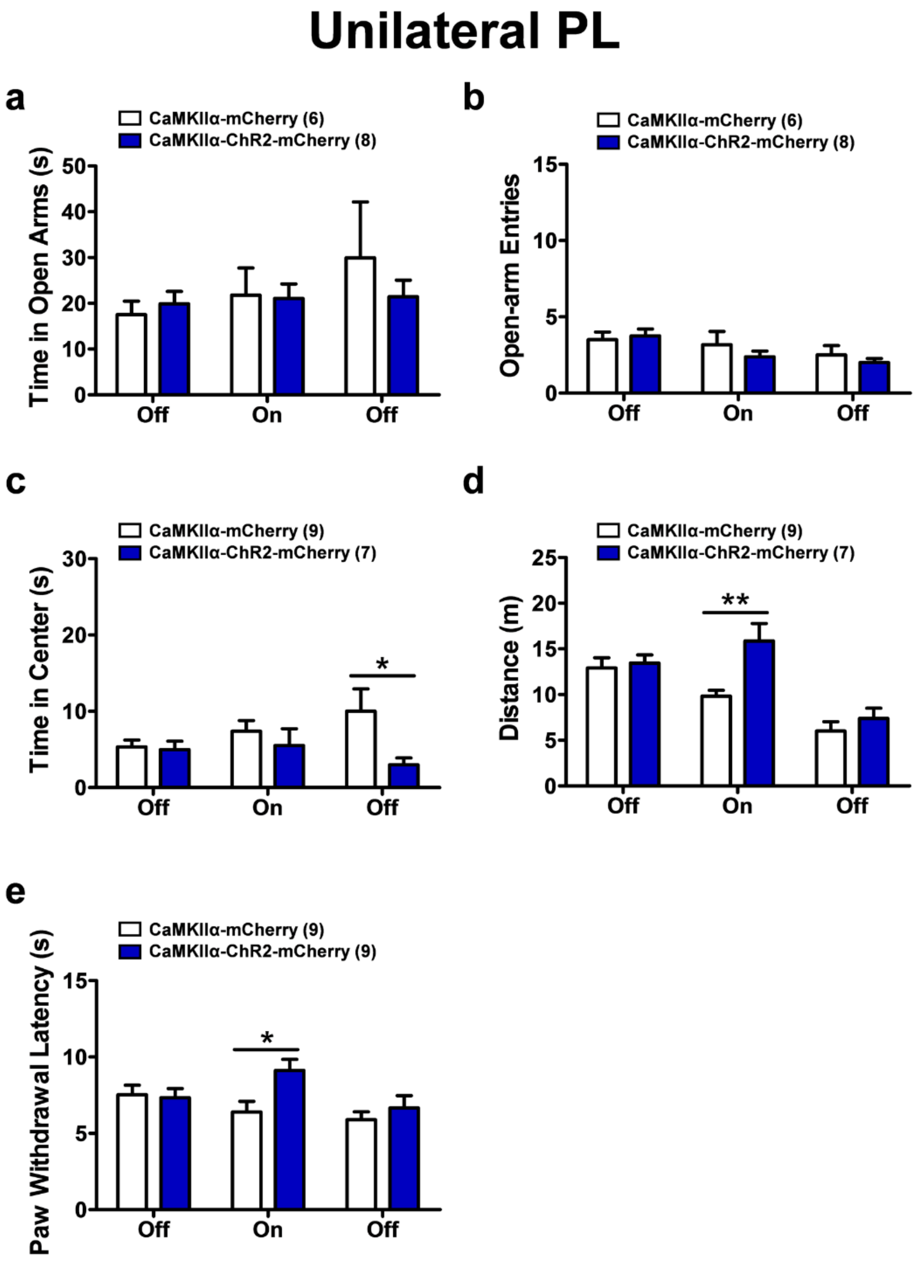

Figure S9. Optogenetic activation of unilateral PL excitatory neurons in na ïve mice increases the paw withdrawal latency without affecting anxiety-like behaviors. Elevated plus maze, open field test, locomotor activity and paw withdrawal latency in the na ïve mice of the AAV-CaMKIIα-mCherry or AAV-CaMKIIα-ChR2-mCherry virus injection with 20 Hz, 6–9 mW 473 nm blue light off-on-off stimulation (**P*<0.05, ***P*<0.01, two-way ANOVA with Bonferroni post-tests).
